# Sex Determination of 3D Skull Based on a Novel Unsupervised Learning Method

**DOI:** 10.1155/2018/4567267

**Published:** 2018-06-25

**Authors:** Hongjuan Gao, Guohua Geng, Wen Yang

**Affiliations:** ^1^College of Information Science and Technology, Northwest University, Xi'an, China; ^2^College of Xinhua, Ningxia University, Yinchuan, China

## Abstract

In law enforcement investigation cases, sex determination from skull morphology is one of the important steps in establishing the identity of an individual from unidentified human skeleton. To our knowledge, existing studies of sex determination of the skull mostly utilize supervised learning methods to analyze and classify data and can have limitations when applied to actual cases with the absence of category labels in the skull samples or a large difference in the number of male and female samples of the skull. This paper proposes a novel approach which is based on an unsupervised classification technique in performing sex determination of the skull of Han Chinese ethnic group. The 78 landmarks on the outer surface of 3D skull models from computed tomography scans are marked, and a skull dataset of a total of 40 interlandmark measurements is constructed. A stable and efficient unsupervised algorithm which we abbreviated as MKDSIF-FCM is proposed to address the classification problem for the skull dataset. The experimental results of the adult skull suggest that the proposed MKDSIF-FCM algorithm warrants fairly high sex determination accuracy for females and males, which is 98.0% and 93.02%, respectively, and is superior to all the classification methods we attempted. As a result of its fairly high accuracy, extremely good stability, and the advantage of unsupervised learning, the proposed method is potentially applicable for forensic investigations and archaeological studies.

## 1. Introduction

Sex analysis and determination are indispensable and foremost steps in confirming the personal identification of an individual in forensic investigations. The best result is achieved when confirming an individual sex by accessing the entire skeleton, but most of the time the skeleton is incomplete. Thus, various local skeletons such as the patella [1], hip joint [2], pelvis [3], calcaneus [4], carpal [5], and skull and its parts have been utilized for sex determination in different populations worldwide. Among all parts of the skeleton, the skull is a small and distinctive collection of bones. The skull is composed of hard tissue and can be well preserved in most cases. Hence, the skull and its parts are most widely and commonly used in providing information about human origin, ancestry, stature, and sex in forensic anthropological analysis [6].

Sex determination of the skull involves two major techniques: the first one is the measurement of skull traits, which reflects difference of skull morphology between males and females. The second one is the analysis and classification of skull measurements. Both will affect the classification accuracy in sex identification for the skull. The approach earlier used to measure skull traits is subjective visual method. Visual assessment depends heavily on the experience and knowledge of the forensic scientist or biological anthropologist. Thus, it is likely to be inaccurate when performed by an inexperienced observer due to its great subjectivity. To reduce subjectivity, efforts to physically quantify skull traits by using an ordinal scale or software are undertaken. With the development and success of medical imaging, skull traits measurement by means of images and computed tomography (CT) is established. For example, some studies used radiograph to provide morphological details of the skull, and some researchers utilized three-dimensional (3D) imaging of the skull from clinical scans of known individuals to discover metric variables. No matter what method is used to measure the morphological features of the skull, it is very important to employ a high-performance classification method. In existing studies, typically statistical and supervised classification methods are linear discriminant analysis (LDA), logistic regression, and support vector machines (SVM).

At present, many approaches in sex determination of the skull, which consist of skull measurement and data classification techniques, have been published and have achieved a high or higher accuracy of discrimination between the sexes. Walker obtained five cranial traits (glabella, mental, orbital, nuchal, and mastoid) by visual assessment and achieved the best classification results of 88% of the modern skulls with a negligible sex bias of 0.1% via the logistic regression model [7]. Robinson and Bidmos selected 230 skull samples from South Africa and extracted 12 measured skull characteristics and got 72.0–95.5 accuracy by establishing five discriminant function equations [8]. Ogawa et al. obtained anthropological measurements of 113 skulls of modern Japanese individuals from forensic anthropological test records. Ten skull measurements were used for statistical analysis, and nine discriminant functions were established. The classification accuracy is between 79% and 93% [9]. Franklin et al. used OsiriX 03 to mark 31 landmarks on 3D skulls of Australian individuals. They calculated a total of 18 linear interlandmark measurements, which were analyzed by discriminant function. The maximum classification accuracy was 90% [10]. Abdel Fatah et al. utilized 222 cranial CT images of White Americans to construct a statistical bone atlas. They obtained >95% accuracy (97.5% with 11 variables and 95.5% with 8 variables) by cross-validated linear discriminant analysis on metric variables [11]. Musilová et al. used coherent point drift-dense correspondence to analyze the entire cranial surface and used an SVM with a radial kernel to perform classification. The method provided a high level of classification accuracy (90.3%) in the sex determination of male and female skulls of Southern French population [12]. Li manually extracted the mid-sagittal frontal arc on dried skulls and adopted the Fourier transform to analyze the sex difference of adult skull in Northeast China. He obtained the results of 84.21% and 83.33% for male and female classification rates, respectively [13]. Li Ming et al. selected 67 skulls from Southwest China and measured 16 anthropometric characters. They established the equations of single-variable and multivariable analysis and obtained the highest accuracy of 89.2% for males and 90.0% for females [14]. Shui et al. chose 133 digital adult skull samples from Han ethnic group of North China and separately computed a total of 14 measurements (12 geometric measurements and 2 angle measurements). Then, they performed the Fisher step method to build the sex discriminant function and obtained the accuracy of 87.5% for male and 86.67% for female separately for the complete skull [15]. Luo et al. constructed a statistical shape model for 208 Chinese skulls by projecting the high-dimensional skull data into a low-dimensional shape space. Fisher discriminant analysis (FDA) was utilized to classify skulls in the shape space; the correct rates were 95.7% and 91.4% for females and males, respectively [16]. Liu et al. divided the skull into seven partitions and quantized immeasurable features by means of marking the feature points. Then, they used the forward stepwise regression method based on maximum likelihood estimation to select the optimal feature subset of each partition. Experiments showed that any three partitions are enough to determine the sex of incomplete skulls with a high accuracy [17].

Although existing methods fully demonstrate their usefulness in sex identification of the skull, a notable problem is that these methods are not applicable in cases in which category labels in the skull samples are absent. Another situation is that when the distribution of the male and female skull samples is not balanced, the effect of using supervised learning for classification may not be better than unsupervised learning. LDA, logic regression, SVM, and other supervised learning methods need to use a training set with category labels to train the classification model. It is therefore the aim of this study to propose a stable and efficient sex determination approach for the skull that is based on unsupervised robust classification technology.

The contribution of our work is as follows: In terms of sex determination of the skull, the current works are mainly focused on the methods of skull measurement, while the methods of data analysis and data classification are less explored, especially the unsupervised learning method. In this study, we attempt to improve the classification accuracy of sex determination of the skull from the perspective of data mining. Inspired by the clustering theories, we extend the fuzzy C-means clustering (FCM) method and put forward an improved algorithm that is used to classify the skull dataset we have measured. We named it as MKDSIF-FCM. The proposed MKDSIF-FCM is based on an unsupervised learning theory where input is presented without desired output. Compared with existing supervised learning methods, the proposed MKDSIF-FCM can divide the skulls into two categories without known category labels and obtain fairly high accuracy for 3D adult skull from the Han Chinese ethnic group.

## 2. Materials and Methods

**Figure 1:**
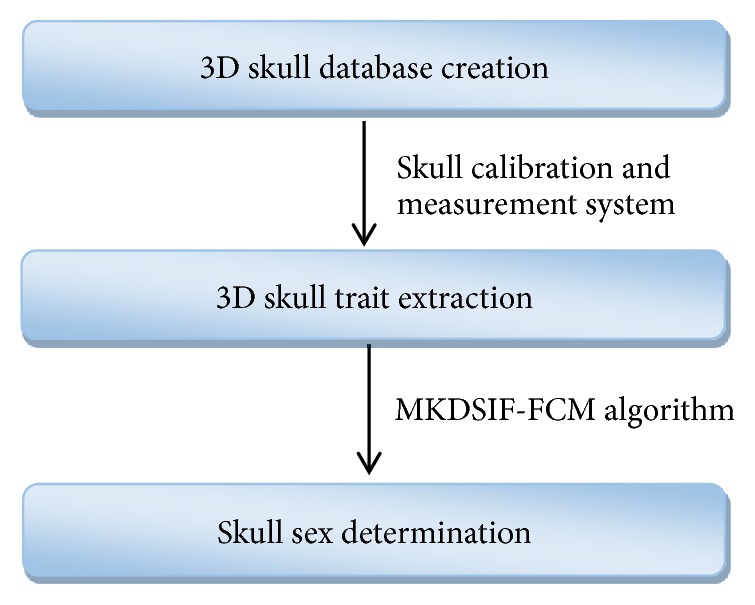
Depiction of the sex determination process.

Our process of sex determination of the skull consists of three broad phases outlined in Figure 1. In the first stage, our approach relies on acquiring skull data and building a database of skull models (Section 2.1). In the second stage, feature points from 3D skull models are marked by utilizing a semi-automatic method, and skull characteristics are extracted that are required to identify a skull (Section 2.2). In the last stage, the extracted characteristics are passed to the classifier. The proposed MKDSIF-FCM algorithm is undertaken to distinguish the skull's sex (Section 2.3).

### 2.1. Skull Data Acquisition

This study is based on the specimens of 186 whole skulls from living adults representative of Han Chinese ethnic group, which were obtained by a clinical multislice CT scanner system at Xianyang Hospital located in Shaanxi province of China. The total database consisted of 100 female skulls with a mean age of 49.8 years (range: 18–75) and 86 male skulls with a mean age of 48.3 years (range: 18–76). Only intact, undamaged skulls were included in this study; each skull contains all the bones from calvaria to jaw with the full mouth of teeth.

### 2.2. Skull Characteristics Measurement

In this study, in order to adequately illustrate the anatomy of the skull, we use the skull calibration and measurement system (with independent research and development by our research group) to extract the characteristics of the 3D skull.

**Figure 2:**
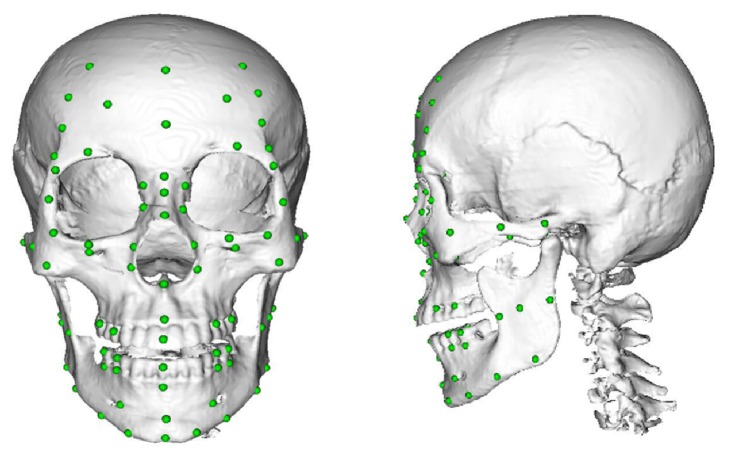
Seventy-eight landmarks on the outer surface of the skull.

According to the research achievements of forensic anthropology experts, 78 landmarks on the outer surface of the skull are marked, 12 of them are located in the midline, and the rest are symmetrically located about the midline sagittal line on both sides (Figure 2).

Distances and angles between different skull landmarks may be important components of skull sexual dimorphism. The size-related variables which reflect sex differences between male and female are obtained by calculating a total of 40 interlandmark measurements. Then, essential characteristic indexes for each skull were successfully constructed.  Table 1 shows the characteristics and their brief descriptions; the data unit is mm.

### 2.3. Method

FCM [19] is an unsupervised learning algorithm and a normal tool for data mining. Clustering is a process for grouping a set of data into classes so that the data within a cluster have high similarity but are very dissimilar to data in other clusters.

To classify our skull measurements via the unsupervised learning method, we propose an improved FCM algorithm that puts forward the concept of distance weighting coefficient with influence factor (IF) and incorporates the advantage of multiple kernel learning. We named it as MKDSIF-FCM.

#### 2.3.1. Distance Weighting Coefficient with IF

In the generic FCM algorithm, *u*_*ik*_ ∈ U is a membership function value from* k*^*th*^ vector* x*_*k*_ to* i*^*th*^cluster center* v*_*i*_. It reflects to what degree the same sample belongs to each cluster center. In (1a)–(1d), there is an example of distance weighting coefficient with IF. (1a)* X* is a set of two-dimensional samples. (1b) *V* represents the initial cluster center in the FCM algorithm. (1c) *U* represents the initial membership function value in the FCM algorithm. (1d) *W*^*β*^ represents the proposed distance weighting coefficient with IF.

In this example, three samples are* a*,* b*, and* c*; two cluster centers are *v*_1_ and *v*_2_. Suppose the membership function values of the sample* a* belonging to *v*_1_ and *v*_2_ are 0.7 and 0.3, respectively. It is obvious that sample* a* belongs to the* v*_*1*_-centered class. The membership function values 0.7, 0.6, and 0.2 could not be compared in generic FCM. Nevertheless, this comparability is very important for classification or clustering analysis. It can reflect the distance of samples* a*,* b*, and* c* to the cluster center *v*_1_.

(1a) X=abc=111244

(1b) V=v1v2=1055

(1c) U=a0.70.3 b0.60.4 c0.20.8cv1v2

(1d) Wβ=715615215315415815β=1

This paper puts forward a new concept of distance weighting coefficient with IF and provides a new approach of distance definition. Distance weighting coefficient is defined according to different contributions of sample to the same cluster center in data space. Distance weighting coefficient with IF is defined as follows:

(2) wi=∑k=1nuikk=0,1,2,⋯,n

(3) wik=uikwi

(4) wik=1wikβ

Let* w*_*ik*_ be a fuzzy weighting coefficient from* k*^*th*^ vector* x*_*k*_ to* i*^*th*^ cluster center* v*_*i*_. Moreover,* w*_*ik*_ plays an important role in measuring the distance between* k*^*th*^ vector* x*_*k*_ and* i*^*th*^ cluster center* v*_*i*_. For different types of sample set, the influence on distance* d*_*ik*_ by* w*_*ik*_ is different. In order to be able to ensure the stable clustering performance of our improved algorithm in regard to different datasets, we introduce an IF for* w*_*ik*_, denoted as *β*.

#### 2.3.2. Euclidean Distance Based on Distance Weighting Coefficient with IF

In generic FCM, Euclidean distance is commonly used as distance *d*_*ik*_. The notion of distance weighting coefficient with IF is introduced by the proposed MKDSIF-FCM algorithm, and the distance from *k*^*th*^ vector* x*_*k*_ to *i*^*th*^ cluster center* v*_*i*_ is defined in the form of square:

(5) $$\begin{array}{c} d_{ik}=\sqrt{{\left(\frac{1}{w_{ik}}\right)}^{\beta }\overset{m}{\underset{j=1}{\sum }}{\left(x_{kj}-v_{ij}\right)}^{2}}={\left\|\;{\left(\;\frac{1}{w_{ik}}\;\right)}^{\beta }\left(\;x_{k}-v_{i}\;\right)\;\right\|}^{2} \end{array}$$

We can prove that (5) obeys with distance definition in Euclidean space. We shall discuss the significance of (1/*w*_*ik*_)^*β*^. In (1a)–(1d), the Euclidean distances of three samples *a*, *b*, and *c* to cluster center* v*_*1*_ are 1, 2, and 5, respectively. Suppose the value of *β* is 1. According to (2), (3), and (4), we can get *w*_11_=7/15, *w*_12_=6/15, and *w*_13_=2/15. According to (5), we can obtain our defined distances of three samples *a*, *b*, and *c* to cluster center *v*_1_: *d*_11_ ≈1.46, *d*_12_ ≈2.24, and *d*_13_ ≈13.69.

From calculating the results, we introduce distance weighting coefficient with IF to distance in Euclidean space, which is equivalent to the function of a zoom lens. It enlarges (*β*≥0) or shortens (*β*<0) all distances, but an enlarged or shortened yardstick is different. For long distances, the enlarged or shortened yardstick is slightly bigger, and for short distances the enlarged or shortened yardstick is slightly smaller. It leads to polarization, in which long distances become much longer, and short distances become much shorter. Thus, an appropriate assignment of distance weighting coefficient with IF can improve the performance of FCM.

#### 2.3.3. Multiple Kernel Learning

In general, the reliability of the traditional clustering algorithms strictly depends on the feature difference of data. If the feature differences are large, it is easy to implement clustering. However, if the feature differences are small and even some features are crossed in the original space, it is difficult for traditional algorithms to cluster correctly. By using the traditional clustering methods and kernel technique, Wu et al. constructed the kernel clustering algorithm [20]. Kernel-based fuzzy clustering can map the data in the original space to a high-dimensional feature space in which it can produce a remarkable improvement over standard FCM. Then, Sonnenburg et al. put forward the concept of multicore learning [21].

The proposed MKDSIF-FCM algorithm incorporates the advantage of multiple kernel learning. Usually, multiple kernel methods consist of polynomial kernel, Gaussian kernel, and hyperbolic tangent kernel. According to different properties of samples, we can choose different parameters of different kernel functions to extend applicability of single kernel function, and we can choose different kernel functions to make the global kernel function and local complementary kernel function, further improving the categorization of different samples. Ultimately, good clustering effect is achieved, and generalization performance of the kernel is improved.

The form of Gaussian kernel function is as follows:

(6) $$\begin{array}{c} K\left(\;m,n\;\right)=\exp\left(\;-\frac{{\left\|m-n\right\|}^{2}}{2\sigma^{2}}\;\right) \end{array}$$

where* n* is the center of kernel function and* σ *is the width parameter and controls the radial range of the function.

The form of polynomial kernel function is as follows:

(7) $$\begin{array}{c} K\left(\;m,n\;\right)={\left(\;m\cdot n+c\;\right)}^{d},\;c\geq 0,\;\;d\in N \end{array}$$

The form of hyperbolic tangent kernel function is as follows:

(8) $$\begin{array}{c} K\left(\;m,n\;\right)=tanh\left(\;-b\cdot \left(\;m\cdot n\;\right)-c\;\right) \end{array}$$

Any function which satisfies the mercer condition [22] can be regarded as a kind of kernel function. The combination of* k* kernel functions according to different weight coefficients is still a kernel function, denoted as the following:

(9) $$\begin{array}{c} K^{*}\left(\;m,n\;\right)=\overset{K}{\underset{k=1}{\sum }}\beta_{k}K_{k}\left(\;m,n\;\right)\;\beta_{k}\geq 0,\;\;k=1,2,\cdots ,K \end{array}$$

Under the constraint,

(10) $$\begin{array}{c} \overset{K}{\underset{k=1}{\sum }}\beta_{k}=1{\;\beta }_{k}\geq 0,\;\;k=1,2,\cdots ,K \end{array}$$

By constraining to the Euclidean distance, the squared distance is computed in the kernel space using multiple kernel functions such that

(11) dki=Φxk−Φvi2=K∗xk,xk+K∗vi,vi−2K∗xk,vi

If we select the Gaussian kernel which is used almost exclusively in the literature, then *k*(*x*, *x*) = 1 and

(12) $$\begin{array}{c} d_{ki}={\left\|\;\Phi \left(\;x_{k}\;\right)-\Phi \left(\;v_{i}\;\right)\;\right\|}^{2}=2-2K^{*}\left(\;x_{k},v_{i}\;\right) \end{array}$$

In this way, the objective function* J*_*S*_ will become the following:

(13) $$\begin{array}{c} J_{S}\left(\;U,V\;\right)=\overset{c}{\underset{i=1}{\sum }}\overset{n}{\underset{k=1}{\sum }}{\left(\;u_{ik}\;\right)}^{S}{\left\|\;\Phi \left(\;x_{k}\;\right)-\Phi \left(\;v_{i}\;\right)\;\right\|}^{2} \end{array}$$

where Φ(.) is the nonlinear map kernel function and Φ(*x*_*k*_) and Φ(*v*_*i*_) express sample* x*_*k*_ and clustering center* v*_*i*_ in feature space, respectively.

Minimizing (13), we then can obtain the update expressions of membership function* u*_*ik*_ and center of cluster* v*_*i*_ as follows:

(14) uik=1−K∗xk,vi−1/s−1∑j=1c1−K∗xk,vj−1/s−1

(15) vi=∑k=1nuiksK∗xk,vixk∑k=1nuiksK∗xk,vi

#### 2.3.4. The Proposed MKDSIF-FCM Algorithm

Assume *X* = {*x*_1_, *x*_2_,…, *x*_*n*_} is a set of *m*-dimensional samples, where *x*_*k*_ = {*x*_*k*1_, *x*_*k*2_,…, *x*_*km*_} represents the* k*^*th*^ sample for* k*=1,2*,..,n* and an integer* c*(2≤*c*≤*n*) is the number of clusters. The* i*^*th*^ cluster is supposed to have the center vector* v*_*i*_= {*v*_*i*1_, *v*_*i*2_,…, *v*_*im*_} (1≤*i*≤*c*).

*U* ∈ *R*_*c*×*n*_ is an* c×n* matrix of fuzzy partition for given training data* x*_*k*_={*x*_*k*1_, *x*_*k*2_,…, *x*_*km*_} (*k*=1,2,…,*n*), where *u*_*ik*_ ∈ *U* is a membership function value from *k*^*th*^ vector* x*_*k*_ to *i*^*th*^ cluster center* v*_*i*_ and *u*_*ik*_ satisfies the following conditions:

(16) ∑i=1cuik=1,∀k

(17) 0<∑k=1nuik<n,∀i

(18) 0≤uik≤1,∀k,i

The MKDSIF-FCM algorithm aims to determine cluster centers* v*_*i*_ (*i*=1, 2,…,* c*) and the fuzzy partition matrix* U* by minimizing the objective function* J*_*S*_ defined as follows:

(19) JsU,V=∑i=1c∑k=1nuiks1wikβ·K∗xk,xk+K∗vi,vi−2K∗xk,vi2

where parameter s(1<*s*<*∞*) influences the fuzziness of the clusters. Large* s* will increase the fuzziness of the function. For most data, 1.5 ≤*s* ≤3.0 gives good results. The value of* s* is often set to 2. Moreover, *d*_*ki*_ is the Euclidean distance of the kernel space from sample* x*_*k*_ to cluster center* v*_*i*_ defined as (11).

The MKDSIF-FCM algorithm uses iterative optimization to approximate minima of an objective function* J*_*S*_. In minimizing* J*_*S*_, the basic step of MKDSIF-FCM algorithm is performed in the following procedures.

**Step 1 .**

Given a value of parameters* c* and commonly in the literature, we let* s*=2.

**Step 2 .**

Initialize the matrix* U* of fuzzy partition by generating* c×n* random numbers in the interval [0,1].

**Step 3 .**

For* t*=0, 1, 2,…, adopt FCM algorithm to calculate cluster centers* v*_*i*_ (*i*=1, 2,…,* c*) by using* U* as follows:

(20) $$\begin{array}{c} v_{i}=\frac{\sum_{k=1}^{n}{\left(u_{ik}\right)}^{S}x_{k}}{\sum_{k=1}^{n}{\left(u_{ik}\right)}^{S}} \end{array}$$

**Step 4 .**

According to (2), (3), and (4), we can obtain *w*_*ik*_.

**Step 5 .**

*U* and* V* are updated by minimizing objective function* J*_*S*_. We can derive the calculating formula of *u*_*ik*_ and* v*_*i*_ as (14) and (15), respectively.

**Step 6 .**

Compute the objective function* J*_*S*_ by using (19); stop the MKDSIF-FCM process if the following condition holds:

(21) $$\begin{array}{c} \left|\;J_{S}\left(\;t+1\;\right)-J_{S}\left(\;t\;\right)\;\right|<\varepsilon \end{array}$$

where it converges or the difference between two adjacent computed values of objective functions* J*_*S*_ is less than the given threshold* ε*.

Otherwise, go to Step 4.

The input of MKDSIF-FCM algorithm is a set of samples* X*={*x*_1_, *x*_2_, …, *x*_*n*_}, and the number of clusters is required to be predefined. Further, two parameters (*s* and* ε*) need to be given in advance. The output of MKDSIF-FCM algorithm are the cluster centers* v*_*i*_ (*i*=1, 2,…,* c*) and the fuzzy partition matrix* U*.

## 3. Results

We use a 3.40 GHZ Core(TM) I7-3770 CPU 4GB RAM desktop computer and MATLAB 2015a software in conducting all experiments. For all algorithms presented in this paper, the experiments were repeated 50 times, and the average results were obtained for comparison.

In MKDSIF-FCM algorithm, there is a parameter group *X* = {*s*, *p*_1,_*p*_2,_*σ*_1,_*σ*_2,_ *β*}, where* s* represents the fuzziness index,* p*_*1*_ and* p*_*2*_ represent the probability,* σ*_*1*_ and* σ*_*2*_ represent the parameters of the Gaussian kernel function, and *β* represents the IF.

For all supervised classification methods presented in this paper, the skull dataset is split into a training set and testing set; 60 samples were randomly picked as the testing set and the numbers of positive and negative examples are kept the same in each sampling.

### 3.1. The Results of Sex Determination for 3D Skulls

The metrics used for evaluating the performance of the algorithm on the skull dataset are described below:

  ACC: it is the number of skulls that are correctly classified as male or female skulls.
  TPR: it is the proportion of the male skulls that are correctly identified.
  TNR: it is the proportion of the female skulls that are correctly identified.
  T: it represents running time.

From Table 2, it can be seen that when selecting a group of suitable parameter values (*s*=2, *β*=0.5,* p*_*1*_=0.9,* p*_*2*_=0.1,* σ*_*1*_=30, and* σ*_*2*_=110), the MKDSIF-FCM algorithm can obtain the best classification accuracy of sex determination of the skull. For 186 skulls of the Han Chinese ethnic group, we obtain the accuracy of 95.70% compared to 87.09%, 92.2%, and 93.55% found in the literature [15–17], respectively. There is a classification accuracy of 93.02% for males and 98% for females, respectively.

### 3.2. Comparison with Other Unsupervised Methods

It is clear from Table 2 that the accuracy had a significant and sharp improvement of nearly 34% for the MKDSIF-FCM algorithm over the original FCM algorithm for the skull dataset. The running time for MKDSIF-FCM is greater than that for FCM, because the number of iterations to convergence is greater.

It is also clear from Table 3 that the MKDSIF-FCM algorithm achieved better classification performance on the Iris dataset. There is an improvement of nearly 6% for MKDSIF-FCM over the original FCM algorithm with detecting a group of suitable parameters. The proposed algorithm appears to have the quite similar time complexity and iterations as the original FCM algorithm.

**Figure 3:**
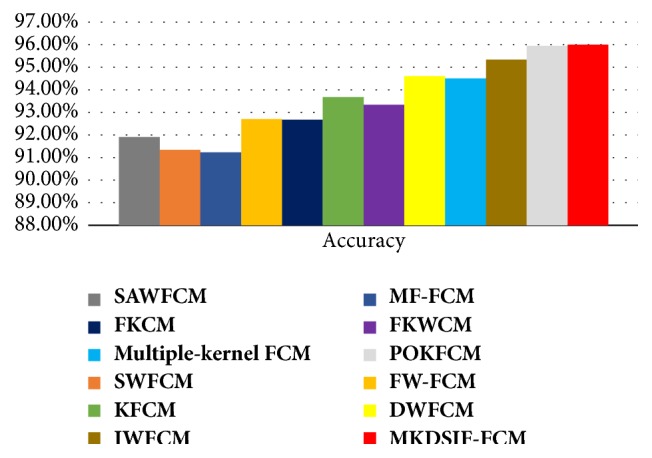
Accuracy analysis of the proposed MKDSIF-FCM and existing improved FCM algorithms on the Iris dataset.

As shown in Figure 3, it is easily observed that the accuracy of MKDSIF-FCM algorithm is higher than that of SAWFCM [23], SWFCM [24], MF-FCM [25], FW-FCM [26], FKCM [27], KFCM [28], FKWCM [29], DWFCM [30], multiple kernel FCM [31], and IWFCM [32]. The accuracy of the MKDSIF-FCM algorithm is quite similar to that of POKFCM [33].

### 3.3. Comparison with Popular Supervised Classification Methods

Table 4 unfolds a clear comparison between the proposed MKDSIF-FCM algorithm and the other six supervised classification methods in three aspects, ACC, TPR, and TNR. All the results we have obtained are as follows (in order of increasing ACC): decision tree (80.47%), BP neural network (83%), H-ELM (88.2%), logistic regression (88.73), SVM (92.8%), FDA (92.87%), and MKDSIF-FCM (95.70%). It is obvious that the proposed MKDSIF-FCM algorithm obtained not only the highest classification accuracy of 95.7% but also the highest TPR and TNR of 93.02% and 98%, respectively. Both FDA (with the best feature) and SVM did a good job with higher accuracy. The classification accuracies of other methods are no more than 90%. The results reveal several similarities between TPR and TNR. And we can observe that the correct classification rate of females is uniformly higher than that of males.

### 3.4. Stability Analysis of the MKDSIF-FCM Algorithm

**Figure 4:**
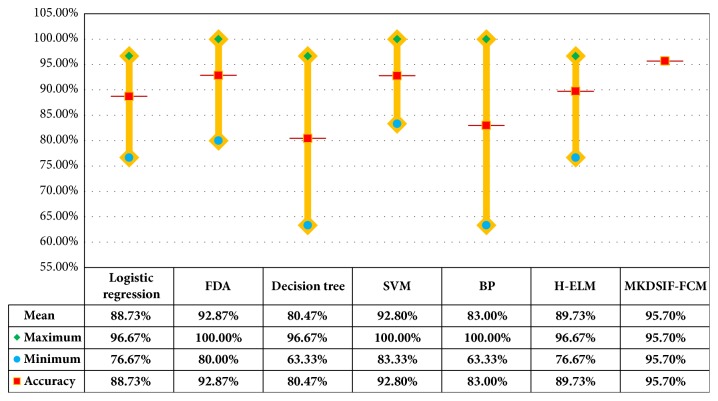
Comparative analysis of stability for the proposed MKDSIF-FCM and other classification methods on the skull dataset.

The experimental procedure is repeated 50 times for each classification method; the maximum, minimum, and mean of the accuracy are represented via error-bar plots (Figure 4). The proposed MKDSIF-FCM algorithm presents an extremely stable performance on the skull dataset, and the classification accuracy of other methods fluctuates greatly. The difference between maximum and minimum accuracy ranged from 37% using BP neural network to 17% for SVM.

## 4. Discussion

FCM [19] is one of the best-known unsupervised algorithms. However, its performance has been limited to Euclidean distance. In recent years, various kinds of improved FCM algorithms have been reported [23–33]. This paper proposes an improved FCM algorithm to determine the sex of adult skulls from the Han Chinese ethnic group. In order to verify the effectiveness and generality of the proposed algorithm, we performed a comparative analysis among the original FCM, some improved FCM algorithms, and the proposed MKDSIF-FCM algorithm.

The MKDSIF-FCM algorithm achieved better classification performance on both publicly available Iris dataset and skull dataset. Especially in the skull database, the accuracy has been greatly improved. On the Iris dataset, our MKDSIF-FCM algorithm has little change in time complexity and iterations compared with FCM. On the skull dataset, the number of iterations of the MKDSIF-FCM algorithm is much larger than that of FCM. This finding implies that the proposed algorithm can tend to become very computationally demanding when the data has high dimensionality and large volume. Experimental results on the Iris datasets show that, for accuracy, our algorithm is almost better than all algorithms in the literature [23–33].

Our innovative algorithm introduces distance-weights with IF into the commonly used Euclidean distance and increases the difference degree of category between samples. The proposed algorithm incorporates the idea of multiple kernel learning that maps the data into a higher-dimensional space in which the nonlinearity fades away and the data become linearly separable. It is the reason that the proposed MKDSIF-FCM algorithm can improve the performance of clustering.

So far, to our knowledge, supervised learning remains the most widely employed method in sex determination of a skull. In particular, logistic regression and discriminant function analysis are the two most representative statistical learning methods. According to the method used in literature [17], we established the best model using logistic regression and stepwise variable selection. When selecting nine variables (I8, I11, I14, I16, I20, I29, I31, I38, I40), the model obtains 84.93% and 92.53% classification rates for males and females, respectively. In the same way, we select the best feature subset from skull measurements to establish the FDA model. With ten variables (I8, I11, I14, I16, I20, I23, I29, I31, I38, I40), the classification rates for males and females are 90.93% and 94.80%, respectively. In order to choose the most suitable classifier for the skull dataset, we also compared the results using other popular supervised classification methods, including decision tree, SVM, BP neural networks, and H-ELM [18]. In all the methods we attempted, the proposed MKDSIF-FCM algorithm gives the best classification performance for both male and female skulls.

When classifying the skull dataset, we hope that the results can be reproduced. Thus, it is very important that the classification algorithm is stable. In the 50 repeated experiments, our algorithm obtained the same result. It is obvious that the proposed MKDSIF-FCM algorithm presents extremely stable performance on the skull dataset.

In conclusion, by means of its fairly high accuracy, extremely good stability, and the advantage of unsupervised learning, we have the reason to believe that the MKDSIF-FCM algorithm is the most suitable classifier for our skull dataset. Of course, our experimental results also indicate that skull characteristics we extracted were very accurate and effective in sex determination of the skull.

## 5. Conclusions

In this paper, we propose a novel approach to sex determination of skulls of the Han Chinese ethnic group. The first step in our method is extraction of morphological features from the 3D skull. In the second step, the MKDSIF-FCM algorithm is employed to conduct sex determination of the skull of the Han Chinese ethnic group. A comparison with other popular classifiers, such as decision tree, BP neural network, logistic regression, FDA, SVM, and H-ELM [18], showed that our proposed MKDSIF-FCM algorithm worked better. The experimental results suggest that the use of the proposed MKDSIF-FCM algorithm in the classification of the skull dataset is an accurate, robust, and reproducible technique. For the Han Chinese ethnic group, there is an accuracy improvement of nearly 8.6%, 3.5%, and 2.2% for our sex determination approach over other methods in the literature [15–17].

It is worth noting that the proposed method achieves a better and stable performance for skull sex determination while maintaining its advantages of unsupervised learning. We believe that the methods described here are noteworthy, particularly for researchers who are attempting (or are considering attempting) to engage in skull sex determination by means of unsupervised learning methods.

## Figures and Tables

**Table 1:** Description of the skull measurements.

Index	Descriptions	Index	Descriptions
I1	Maximum cranial length	I21	Maximum breadth of the frontal bone
I2	Basicranial length	I22	Upper facial width
I3	Maximum cranial breadth	I23	Orbital width
I4	First cranial height	I24	Width of the superior alveolar arch
I5	Bizygomatic breadth	I25	Length of the maxillary alveolar arch
I6	Bimaxillary width	I26	Palatal length
I7	Upper facial height	I27	Palatal width
I8	Minimum frontal breadth	I28	Palatal height
I9	First orbital width of the right eye	I29	Bigonial breadth
I10	Second orbital width of the right eye	I30	Height of the mandibular joint
I11	Distance between the outer corners of both eyes	I31	Height of the right mandibular ramus
I12	Height of right eye	I32	Mandibular condylar width
I13	Distance between the inner corners of the both eyes	I33	Coracoid width
I14	Nasal height	I34	Width of the mandibular notch
I15	Height of the nose forehead	I35	Depth of the mandibular notch
I16	Nasal width	I36	Height of the mandibular ramus
I17	Right mastoid length	I37	Thickness of the mandibular body
I18	Bimastoid width	I38	Mandibular angle
I19	Distance from the occipital to right mastoid point	I39	Second cranial height
I20	Frontal string	I40	Superciliary arch

**Table 2:** Comparative analysis of the proposed MKDSIF-FCM and original FCM algorithms on the skull dataset.

Result	FCM	MKDSIF-FCM
	s=2	s = 2 *β* = 0.5 p_1_ = 0.9 p_2_ = 0.1 *σ*_1_ = 30 *σ*_2_ = 110
TPR [%]	28.00	98.00
TNR [%]	100.0	93.02
ACC [%]	61.29	95.70
T[s]	0.0074	0.1281
Iterations	17	102

**Table 3:** Comparative analysis of the proposed MKDSIF-FCM and original FCM algorithms on the Iris dataset.

Result	FCM	MKDSIF-FCM
	s=2	s = 2 *β* = 0.5 p_1_ = 0.7 p_2_ = 0.3 *σ*_1_ = 3 *σ*_2_ = 0.5
Accuracy [%]	89.33	96.00
T [s]	0.0042	0.0145
Iterations	16	11

**Table 4:** Comparative analysis of the proposed MKDSIF-FCM and other popular classification methods on the skull dataset.

Classifier	TPR [%]	TNR [%]	ACC [%]
Decision tree	78.82	82.10	80.47
BP neural network	80.93	85.07	83.00
H-ELM [18]	88.27	88.13	88.20
Logistic regression	84.93	92.53	88.73
SVM	91.60	94.00	92.80
FDA	90.93	94.80	92.87
MKDSIF-FCM (proposed)	93.02	98.00	95.70

## Data Availability

The data used to support the findings of this study are available from the corresponding author upon request.
